# Self-reported quantity and quality of sleep in children and adolescents with a chronic condition compared to healthy controls

**DOI:** 10.1007/s00431-023-04980-8

**Published:** 2023-04-26

**Authors:** Camille F. M. Biemans, Sanne L. Nijhof, Jan Willem Gorter, Gonneke J. W. M. Stevens, Elise van de Putte, Johanna W. Hoefnagels, Anemone van den Berg, Cornelis K. van der Ent, Jeroen Dudink, Olaf W. Verschuren

**Affiliations:** 1 Center of Excellence for Rehabilitation Medicine, University Medical Center (UMC) Utrecht Brain Center, UMC Utrecht, Utrecht University (UU) and De Hoogstraat Rehabilitation, Utrecht, The Netherlands; 2 Department of Pediatrics, Wilhelmina Children’s Hospital, UMC Utrecht, UU, Utrecht, The Netherlands; 3 Department of Rehabilitation, Physical Therapy Science & Sports, UMC Utrecht Brain Center, UMC Utrecht, Utrecht, the Netherlands; 4 Department of Interdisciplinary Social Sciences, Faculty of Social and Behavioral Sciences, Utrecht University, Utrecht, The Netherlands; 5 Department of Neonatology, Wilhelmina Children’s Hospital, UMC Utrecht, Utrecht, The Netherlands; 6 Department of Pediatric Pulmonology, Wilhelmina Children’s Hospital, UMC Utrecht, UU, Utrecht, The Netherlands; 7 Department of Pediatric Gastroenterology, Wilhelmina’s Children Hospital/UMC Utrecht, Utrecht, The Netherlands

**Keywords:** Pediatrics, Adolescent, Sleep, Sleep quality, Medically unexplained symptoms

## Abstract

To assess self-reported quantity and quality of sleep in Dutch children with a chronic condition compared to healthy controls and to the recommended hours of sleep for youth. Sleep quantity and quality were analyzed in children with a chronic condition (cystic fibrosis, chronic kidney disease, congenital heart disease, (auto-)immune disease, and medically unexplained symptoms (MUS); n = 291; 15 ± 3.1 years, 63% female. A subset of 171 children with a chronic condition were matched to healthy controls using Propensity Score matching, based on age and sex, ratio 1:4. Self-reported sleep quantity and quality were assessed with established questionnaires. Children with MUS were analyzed separately to distinguish between chronic conditions with and without an identified pathophysiological cause. Generally, children with a chronic condition met the recommended amount of sleep, however 22% reported poor sleep quality. No significant differences in sleep quantity and quality were found between the diagnosis groups. Children with a chronic condition and with MUS slept significantly more than healthy controls at ages 13, 15, and 16. Both at primary and secondary school, poor sleep quality was least frequent reported in children with a chronic condition and most often reported in children with MUS.

*Conclusion:* Overall, children with chronic conditions, including MUS, met the recommended hours of sleep for youth, and slept more than healthy controls. However, it is important to obtain a better understanding of why a substantial subset of children with chronic conditions, mostly children with MUS, still perceived their sleep quality as poor.

**Table:** 

**What is Known:** *• According to the Consensus statement of the American Academy of Sleep medicine, typically developing children (6 to 12 years) should sleep 9 to 12 h per night, and adolescents (13 to 18 years) should sleep 8 to 10 h per night.* *• Literature on the optimal quantity and quality of sleep in children with a chronic condition is very limited.*	
**What is New:** Our findings are important and provide novel insights: *• In general, children with a chronic condition sleep according to the recommended hours of sleep.* *• A substantial subset of children with chronic conditions, perceived their sleep quality as poor. Although this was reported mostly by children with medically unexplained symptoms (MUS), the found poor sleep quality was independent of specific diagnosis.*

**What is Known:**

*• According to the Consensus statement of the American Academy of Sleep medicine, typically developing children (6 to 12 years) should sleep 9 to 12 h per night, and adolescents (13 to 18 years) should sleep 8 to 10 h per night.*

*• Literature on the optimal quantity and quality of sleep in children with a chronic condition is very limited.*

**What is New:**

Our findings are important and provide novel insights:

*• In general, children with a chronic condition sleep according to the recommended hours of sleep.*

*• A substantial subset of children with chronic conditions, perceived their sleep quality as poor. Although this was reported mostly by children with medically unexplained symptoms (MUS), the found poor sleep quality was independent of specific diagnosis.*

## Introduction

Over the past decades, average sleep duration worldwide has decreased in children [1]. Children currently sleep more than 1 h (h) less per night compared to children in the twentieth century [1]. This is alarming, since adequate sleep, both in quantity and quality, is important for childrens’ health and development [2]. In children who attend primary school (8–12 years), sleep is crucial for the development of physical, cognitive and social performance such as learning, repair and memory consolidation [3]. The period at secondary school (12–19 years) is considered a vulnerable period for sleep due to the significant changes in physiology children go through [4]. These changes cause a shift in circadian rhythms and are accompanied by later onset of tiredness which, combined with early school hours, might lead to sleep deprivation [4].

Numerous studies have been performed on recommendations for the optimal sleep quantity in typically developing children [5–8]. According to the Consensus statement of the American Academy of Sleep medicine, children (6 to 12 years) should sleep 9 to 12 h per night, and adolescents (13 to 18 years) should sleep 8 to 10 h per night [7]. In contrast, literature on the optimal quantity and quality of sleep in children with a chronic condition is very limited. Due to condition-related disturbances, such as pain, rigidity, breathing problems or incontinence, children with a chronic condition are expected to have more problems regarding sleep than typically developing peers [9]. However, it is still unclear how these children with a chronic condition perceive their sleep quality.

Within the group of children with a chronic condition, children with medically unexplained symptoms (MUS) form a group that requires a specific approach. These children suffer from physical pain or another physical symptom that cannot be explained by an underlying pathophysiological cause [10]. A disturbed perception of body signals is part of the symptom complex of MUS [11]. Because of these illness-related disturbed perceptions it is interesting to explore how this group perceives their own sleep quantity and quality relative to the rest of the group with a chronic condition in our study.

This exploratory study focuses on sleep data from two existing datasets of children with a chronic condition, including MUS, and of healthy controls. The objective of this study is to determine the self-reported quantity and quality of sleep in Dutch children (8–19 years old) with a chronic condition, as compared to healthy controls, and compared to the recommended amount of sleep [7].

## Methods

### Design

This study is a cross-sectional convenience sample from a cohort study in children with a chronic condition (8–19 years old) and a convenience population sample of healthy controls (11–17 years old).

#### Children with a chronic condition

Data for children with a chronic condition (number (n) = 291) were obtained via the ongoing Patient Reported Outcomes in Children and children with Chronic/life-threatening conditions and Tailored InterVentions in a digital Environment cohort (PROactive cohort) [12]. The response rate was 72% [12]. Detailed information about the PROactive cohort study can be found elsewhere [12, 13]. The PROactive study was classified by the institutional review board (Medical Ethics Review Committee of the University Medical Center Utrecht, The Netherlands), to be exempt from the Medical Research Involving Humans Act (WMO), case number METC 16/707-C, and adhered to all local laws and the Declaration of Helsinki.

#### Healthy controls

Reference data for healthy controls were obtained via the Health Behavior in School-aged Children (HBSC) study [14]. HBSC is a cross-sectional, school-based survey with a focus on health and wellbeing that is conducted every four years in over 40 countries among nationally representative samples of children. The present study made use of the Dutch data collected in 2017 (n = 8306) [14]. The response rate was 39% and 37% for primary and secondary school, respectively [15]. More detailed information about the methodology of the HBSC study can be found in the study protocol [15].

All children with a chronic condition and the same age as children in the HBSC sample, were matched to healthy controls using a 1:4 ratio and Propensity Score Matching with Optimal Matching [16]. Matching was done based on age and sex, as these are important factors influencing sleep and were variables that were collected in both convenient samples [17, 18]. Matching was considered successful if age and sex did not differ significantly between children with a chronic condition and their healthy peers, as determined by independent samples T-tests.

### Study sample

#### Children with a chronic condition

Participants were included as part of the PROactive cohort, for which data collection started in December 2016 and is still ongoing [12]. Inclusion criteria are: children aged 8 to 19 years at time of assessment, with the following diagnoses: cystic fibrosis (CF), chronic kidney disease (CKD), congenital heart disease (CHD) or (auto-)immune disease (AID). All participants were patients at the Wilhelmina Children’s Hospital, Utrecht, the Netherlands. As determined by the treating physician, all participants were in stable stage of their chronic condition (assessment took place at least one year after diagnosis) [12].

Children with medically unexplained symptoms (MUS) formed a distinct group of participants from the PROactive cohort. Children with MUS were included, analyzed and reported separately in order to be able to distinguish between sleep in children with a chronic condition with or without an identified pathophysiological cause. Exclusion criteria for all children with a chronic condition were: cognitive functioning below the level of an eight-year-old child, the inability to understand or read Dutch, the inability to fill out online questionnaires.

Children aged 8–11 years old were assisted by their parents, children 12 years and older filled out the questionnaires individually. All children were asked to fill out a questionnaire about sleep once.

#### Healthy controls

Sleep related data for healthy controls (11–17 years) were collected from the HBSC database [14]. Children filled out questions from the HBSC protocol that are related to sleep quantity and quality under supervision of a trained research assistant [15]. Because some of these children might have underlying conditions that influence the results, healthy controls who reported that they had a long-term (> 3 months) mental or physical condition or disability were excluded from the analysis. This was done to ensure that the health controls did not include children with a chronic condition.

### Outcome measures

#### Self-reported sleep quantity

Self-reported sleep quantity was measured by ‘sleep duration’ in hours as calculated by ‘the moment you closed your eyes and started sleeping’ minus ‘the moment you woke up’. This information was derived from HBSC sub-questions, according to the HBSC protocol [15].

For each age, sleep quantity was compared to the recommended hours of sleep according to the Consensus statement of the American Academy of Sleep Medicine [7].

#### Self-reported sleep quality

Self-reported sleep quality was measured with five items about sleep retrieved from the Groningen Sleep Quality Scale, with an acceptable Cronbach’s alpha of 0.71 [19, 20]. Items were: ‘I feel that I slept poorly’, ‘It took me more than half an hour to fall asleep, ‘I feel that I didn’t get enough sleep’, ‘After I woke up, I had trouble falling asleep again’ and ‘I felt rested after waking up in the morning’. Questions were answered using a 5-point liker scale (1 = never, 2 = almost never, 3 = occasionally 4 = often 5 = (almost) always). The latter item, ‘I felt rested after waking up in the morning’, required recoding in order to be scaled in the same direction as the other items (i.e. a higher score indicates worse self-reported sleep quality). Finally, the average score of the five items was calculated. A self-reported sleep quality score of > 3.5 was considered poor sleep quality [14].

All sleep quantity- and quality-related items were asked about the previous week (Sunday to Thursday). Since sleep rhythms are known to be irregular in children and may differ too much from the child’s typical sleep rhythm during the week, only weekdays were included in the analysis [21, 22].

### Data analysis

All statistical analyses were performed using SPSS for Windows, Version 27.0 (IBM Corp. Released 2017. IBM SPSS Statistics. Armonk, NY: IBM Corp.). To determine normality of the data, Shapiro–Wilk tests were used. Normally distributed data were shown as mean ± standard deviation (SD), frequencies as number (n), %, and non-parametric data as median ± interquartile range (IQR). A p-value of less than 0.05 was considered statistically significant. To identify the missing data pattern, Little’s Missing Completely at Random (MCAR) test was used. Patients who had non-random missing data for sleep quantity and quality variables were excluded from analysis.

We first examined all completed self-reported sleep quantity and quality solely in the PROactive dataset. Data of children with a chronic condition were divided into primary school (up to 12 years) and secondary school children (13–19 years). This cut-off value was chosen because HBSC uses the same cut-off age value [15].

Descriptives were used to compare the sleep quantity of children with a chronic condition with the Consensus statement of the American Academy of Sleep Medicine [7]. One-way ANOVA with Tukey’s post-hoc testing was used to analyze differences in sleep quantity and quality between diagnosis groups.

Secondly, children with a chronic condition were matched to healthy controls. Analyses were performed between children with a chronic condition other than MUS, children with MUS, and healthy controls. One-way ANOVA with Tukey’s post-hoc testing was used to analyze differences in sleep quantity and quality between children with a chronic condition, MUS and healthy controls. Descriptives were used to present sleep quality as well as calculation of the percentage with poor/good sleep quality (using the definition mentioned above) for each group. Differences for subgroups (diagnosis groups and age), were analyzed using one-way ANOVA with Tukey’s post-hoc testing.

## Results

### Children with a chronic condition

In the PROactive cohort, 291 children with a chronic condition were analyzed in this study; no values for patient characteristics were missing. Sleep data were missing completely at random (χ2 (2) = 1.222 *P* = *0.543*). Among all children with a chronic condition (n = 291), children with MUS represented the largest group (n = 111), followed by children with AID (n = 108), CF (n = 45), CHD (n = 19) and CKD (n = 8).

#### Self-reported sleep quantity

**Fig. 1:**
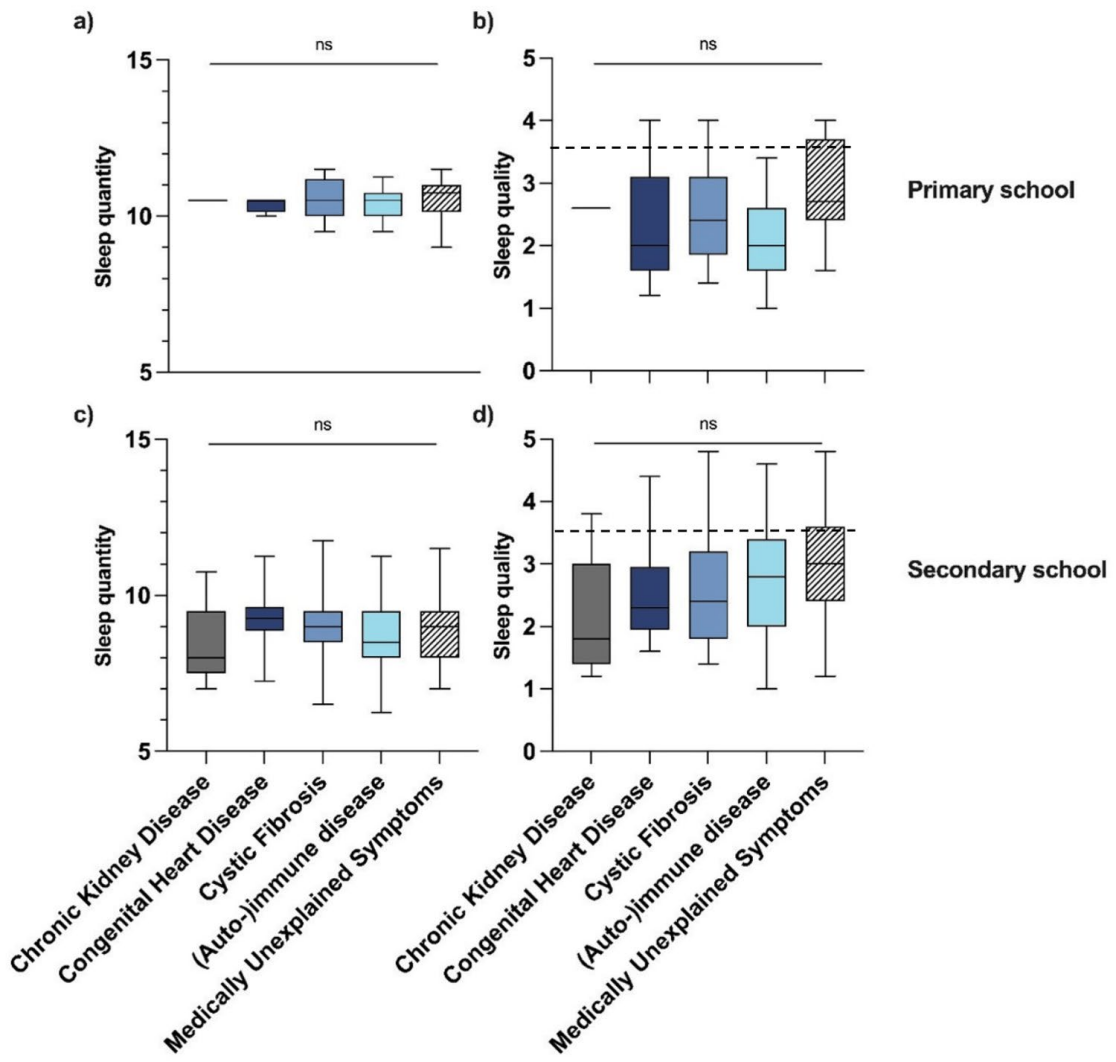
Sleep **a** quantity and **b** quality of children with a chronic condition (n = 291) in primary school and sleep **c** quantity and **d** quality of children with a chronic condition in secondary school, per diagnosis group. A higher score indicates a poorer sleep quality; the dashed line indicates the cut-off point for poor sleep quality (> 3.5). Abbreviations: *ns* non-significant

On average, the self-reported sleep quantity of children with a chronic condition in primary and secondary school was 10:29 ± 0:33 h and 8:53 ± 0:9 h, respectively. In primary and secondary school, no significant differences were found between the diagnosis groups (*P* = *0.793* and *P* = *0.329*, respectively), Fig. 1a, c.

#### Self-reported sleep quality

Of the children with a chronic condition, 22% reported poor sleep quality. For children in primary and secondary school this percentage was 9.4 and 25, respectively.

In primary school, no significant differences were found between the diagnosis groups (*P* = *0.128*), Fig. 1b*.* In secondary school, post-hoc testing revealed that diagnosis groups did not differ significantly from each other, Fig. 1d*.*

The poorest sleep quality has been found in the MUS group both in primary and secondary school, with an average sleep quality of 2.88 ± 0.81 and 3.04 ± 0.86, respectively, Fig. 1b, d.

### Children with a chronic condition compared to MUS and healthy controls

Of the 291 children with a chronic condition, a total of 171 children were the same age as children in the HBSC sample, and thus were found to be eligible to be matched to healthy controls (n = 684). Matching was successful (*P* = *0.754* for sex and *P* = *1.000* for age); see for baseline characteristics Table 1*.*

**Table 1:** Characteristic, sleep quantity and quality of eligible children with a chronic condition without children with MUS, children with MUS and matched healthy controls

	**Chronic condition**		**Medically unexplained symptoms**		**Healthy controls**		P-value*
	Primary school	Secondary school	Primary school	Secondary school	Primary school	Secondary school	
	*(n *= *22)*	*(n* = *92)*	*(n* = *7)*	*(n* = *50)*	*(n* = *174)*	*(n* = *510)*	
**Sex*** (n female, %)*	10, 46	51, 55	5, 71	34, 68	91, 48	318, 62	0.754
**Age*** (mean* ± *SD)*	10.8 ± 1.1	14.5 ± 1.6	11.0 ± 1.3	14.8 ± 1.3	11.1 ± 1.1	15.0 ± 1.2	1.000
**Sleep quality** *(n poor quality, %)*	2, 9.1	16, 17	2, 29	12, 24	35, 20	111, 22	0.260^a^ 0.142^b^
**Diagnosis group**					N.A.	N.A.	
Cystic fibrosis* (n, %)*	8, 28	18, 13					
(Auto-)immune disease* (n, %)*	10, 35	61, 43					
Congenital heart disease*(n, %)*	4, 14	11, 7.7					
Chronic kidney disease *(n, %)*	0, 0	2, 1.4					
Medically unexplained symptoms* (n, %)*			7, 24	50, 35			

*n* number*, SD* standard deviations*, h* hour*, min* minute

*Between children with a chronic condition, MUS and healthy controls, of which ^a^in primary school and ^b^in secondary school

#### Self-reported sleep quantity

In primary school, the average sleep quantity of children with a chronic condition, MUS and healthy controls were 10:25 ± 0:28 h, 10:32 ± 0:49 h and 9:56 ± 0:55 h, respectively, see Table 1*.* In secondary school, children with a chronic condition, MUS and healthy controls slept on average 9:02 ± 0:01 h, 9:04 ± 0:58 h and 8:23 ± 0:53 h, respectively, see Table 1*.*

**Fig. 2:**
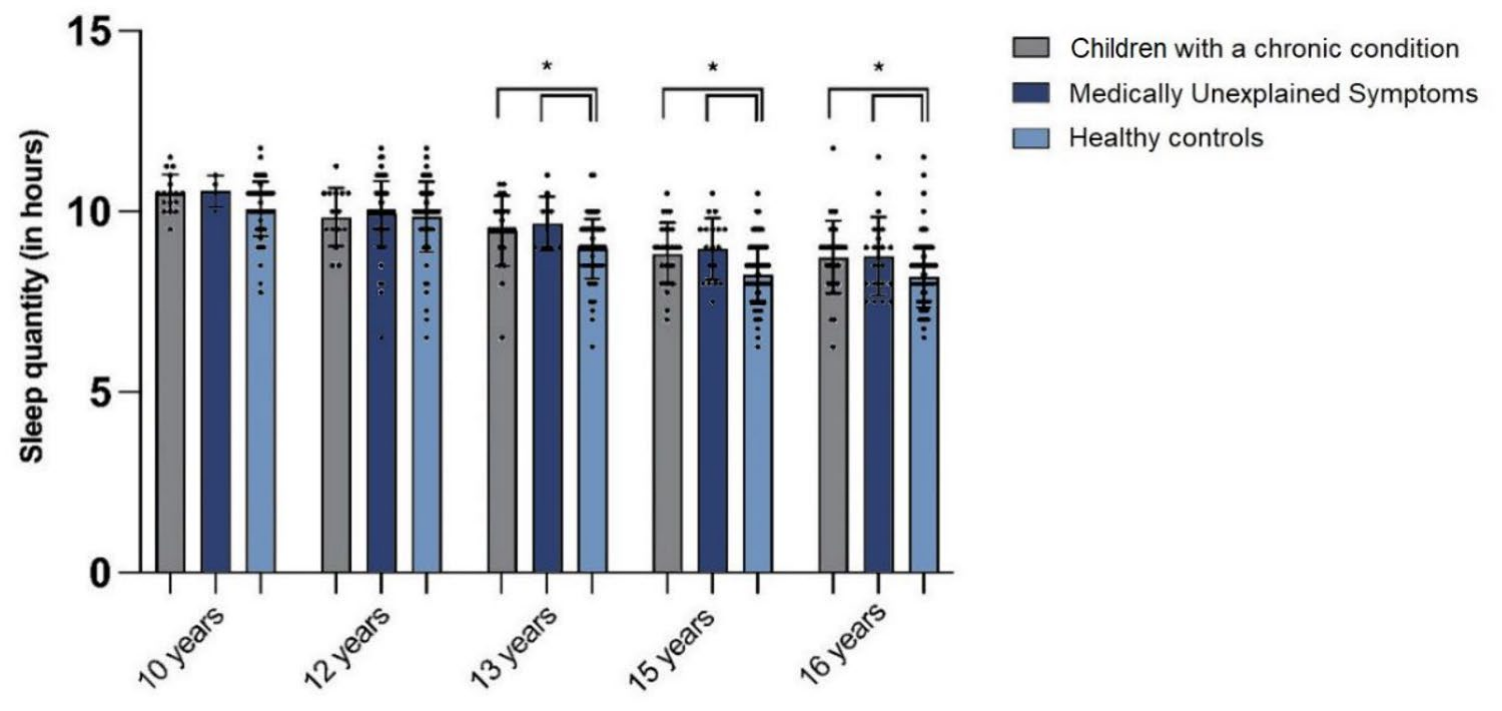
Self-reported sleep quantity in children with a chronic condition without children with MUS, children with MUS and matched healthy controls, per age. *P ≤ 0.05

At the ages of 10, 13, 15, and 16, self-reported sleep quantity of children with a chronic condition differed significantly between children with a chronic condition, MUS, and healthy controls (*P* = *0.049, P* = *0.005, P* = *0.000, and P* = *0.000*, respectively, illustrated in Fig. 2*.* Children with a chronic condition slept significantly more than healthy controls at the ages of 13, 15, and 16 (*P* = *0.033, P* = *0.001, and P* = *0.003,* respectively), see Fig. 2. At the same ages, children with MUS slept more than healthy controls (*P* = *0.048, P* = *0.003,* and *P* = *0.017, respectively),* see Fig. 2*.*

#### Self-reported sleep quality

The percentages that reported poor sleep quality in primary school were 9.1 in children with a chronic condition, 29 in children with MUS and 20.6 in healthy controls. In secondary school, these percentages were 17 among children with a chronic condition, 24 in children with MUS and 22 in healthy controls, see Table 1. Self-reported sleep quality did not differ significantly between children with a chronic condition, MUS, and healthy controls, both in primary and secondary school ages/levels (*P* = *0.260* and *P* = *0.142*, respectively, see Table 1).

## Discussion

In this exploratory study, we aimed to add on our knowledge of the quantity and quality of sleep in children with and without a chronic condition. The study demonstrated that in general, children with a chronic condition slept according to the generally recommended sleep guidelines for typically developing peers [7]. Children with a chronic condition and MUS slept longer than healthy controls. Although a considerable part of all children reported poor sleep quality, children with MUS most often reported poor sleep quality.

### Sleep quantity

When solely looking at sleep duration in children with a chronic condition, in general all children met the recommended hours of sleep per night [7]. Children with MUS slept the longest, and children with a chronic condition slept more than healthy controls. This result is surprising as several studies have shown that various elements of chronic conditions have a bidirectional relationship with diminished sleep quantity [23, 24]. However, since disease activity was generally low in patients included in the PROactive cohort, these biological disease-related symptoms may not have led to diminished sleep quantity in this group [12]. Furthermore, Cohodes et al. showed that a higher anxiety level was linked to a higher sleep quantity, which might be explanatory for the longest sleep duration in children with MUS [25].

Next to that, healthy controls had the shortest sleep duration. In a recent study in school-going typically developing children in Germany, there was shown that only 10% of the German children slept according to the recommended hours of sleep [26]. Not adhering to physical activity and screen time guidelines were the primary contributing factors for this.

Although the majority of children slept according to generally recommended guidelines, one should keep in mind that these guidelines are not specified for children with a chronic condition. Self-reported sleep quality provides important information about how these children experienced their sleep.

### Sleep quality

In general, approximately a quarter of the children with a chronic condition reported poor sleep quality. It is remarkable that the lowest percentage reporting poor sleep quality was amongst children with a chronic condition. Although previous research showed limited sleep quality in CKD and other diagnostic groups, such as heart failure, our results did not confirm this [27, 28]. However, these discrepancies might be related to differences in disease activity and age between our study and the aforementioned studies, that were done in older populations with a higher disease activity [27, 28].

Our results confirmed that children with MUS most frequently reported poor sleep quality. A recent review that looked at the relationship between MUS and sleep disorders, reported that discomfort resulting from experienced somatic symptoms could influence dopamine and endogenous opioid signaling, and thereby affect sleep patterns [29]. Further, in a study where sleep deprivation was induced, a direct link was found between musculoskeletal pain and non-REM sleep deprivation [30].

As well as these possible physiological explanations, psychosocial variables may also play a role. Psychosocial factors, such as social disruption, stress or excessive worrying, are important contributors to onset, persistence, severity, and consequences of MUS [31]. It is known that children with MUS are more likely to suffer from a distorted perception of physical symptoms [11]. For that reason, a possible explanation for the poor sleep quality found in MUS might be that self-reported sleep quality is reflected by distorted perception in this group of children.

### Limitations

When interpreting these results, some limitations should be taken into account. Firstly, since we worked with two convenience samples, research possibilities were limited. No other possible contributing factors could be added to the analysis (for example, medication usage, sleep problems or frequency of acute illness exacerbations). Besides, not all chronic conditions, such as neurological conditions, were included in the PROactive cohort. Therefore, the results should be interpreted as an exploratory study.

It is a limitation that there has not been made use of standardized sleep questionnaires or objective sleep measurement. Debate exists about how to optimally measure sleep, and combining subjective and device-based measurements is recommended [32]. Actual time asleep is often less than time spent in bed, therefore overestimation of the actual time asleep could have been made [33]. However, since children in both cohorts answered the exact same questions about sleep, the differences found between these groups are presumed to reflect valid differences. Most importantly, self-reported data provide unique information about someone’s own perceptions of sleep quantity and quality, which in this case might be more valuable and insightful than objective data.

Lastly, in this study there was only made use of sleep data during week days. There is known that especially sleep quantity substantially differs between week and weekend days [21]. To be able to address the total sleep pattern, weekend days should be included in future research.

### (Clinical) implications

It is important to obtain a better understanding of why children with chronic conditions slept more than healthy controls, and yet a substantial subset of children with chronic conditions, mostly children with MUS, still perceived their sleep quality as poor. In general, more attention to sleep in children with a chronic condition is advised, including monitoring sleep as part of standard care, and a personalized approach which focusses on the individual biopsychosocial factors that play a role in sleep problems. The ultimate goal is a better understanding of sleep requirements of children with a chronic condition and provide them with optimal support.

## Abbreviations

AID: (Auto-) immune disease
CF: Cystic fibrosis
CHD: Congenital heart disease
CKD: Chronic kidney disease
HBSC: Health Behavior in School-aged Children
h: Hour
IQR: Interquartile range
MCAR: Little’s Missing Completely at Random
METC: Medical Ethics Review Committee
MUS: Medically unexplained symptoms
n: Number
PROactive cohort: Patient Reported Outcomes in Children and children with Chronic/life-threatening conditions and Tailored InterVentions in a digital Environment cohort
SD: Standard deviation
UMC: University medical center
UU: Utrecht University
WMO: Medical research involving humans act
